# 

**Published:** 1918-02-09

**Authors:** 


Rates of Subscription: Twelve months, 6/6; Six months, 4/-; three months, 2/-, post free to any address in the United Kingdom. Abroad, Twelve months, 8/8; Six months, 5/-; Three months, 2/6, post free. **THE HOSPITAL "Index" to Volume LXII., April 1917 to September 1917, is now ready, price 2½d. per copy, post free.** Address The Manager, The Hospital, "The Hospital" Building, 28/29 Southampton Street, Strand, London, W.C. 2.

### Gifts and Bequests.

Mr. John William Roberts, of Hale, left the whole of his estate on the death of his wife for charitable purposes, the bequests including £1,000 for Manchester Royal Infirmary.

The Queen has sent donations of £10 each to the funds of Queen Charlotte's Lying-in Hospital, the Royal Dental Hospital, Leicester Square, and the City of London Hospital for Diseases of the Chest (Victoria Park).

Sir Leonard Llewelyn and the Llewelyn family have contributed 1,000 guineas to the Royal Gwent Hospital, Newport, for the endowment of a bed. Mr. Lewis Haslam, M.P., has sent 100 guineas towards wiping off the adverse balance on the hospital's account.

Mr. W. W. Hewitt, of Dedham, near Colchester, who died on September 11 last, left £10,000, on the death of his sister, to the Essex County Hospital, and, subject to other legacies, the residue of his estate of £67,000 to charitable objects selected by his executors.

Under the will of Mr. Edward Sturdy, of Teddington, £500 is left to the Royal Victoria Hospital, Bournemouth, £200 each to the London Hospital and Queen Mary's Convalescent Hospital, Roehampton, and £105 each to the Imperial Cancer Research Fund and the Star and Garter Hospital, Richmond.

Sir John Reynolds Roberts, of Woodford Green, left substantial legacies to textile trade institutions, £4,000 to the West Ham Hospital for the endowment of a ward, £3,000 each to the Victoria Park Hospital for Consumption and the Y.M.C.A., £2,000 each to the Cancer Hospital and the Hospital for Women, Soho Square, £1,500 to the Surgical Aid Society, and £1,000 to the East London Hospital for Children.

Messrs. Barnato Bros., in memory of Mrs. Kate Joel, have made a number of donations, including Middlesex Hospital, £1,050; Westminster Hospital, £1,000; King George's Fund for Sailors, £250; Royal Dental Hospital, £105; and Lord Mayor Treloar Cripples' Hospital, £50.

Miss Talbot, of Margam Abbey, has given £30,000 for the foundation of a chair of preventive medicine at the Welsh Medical School, Cardiff.

The Royal Dental Hospital, Leicester Square, has received a donation of £250, being the amount allocated to the hospital by the trustees of the late Leopold Salomons, Esq.

Mr. Alexander Loughnan, of West Dorking, left £1,000 to the Hospital of St. John and St. Elizabeth, Hampstead.

Several Liverpool institutions benefit under the will of the late Mr. T. Bellringer Howarth; they include £100 each to the Royal Infirmary, the Bluecoat Hospital, the David Lewis Northern Hospital, and the Southern Hospital, and £50 to the Infirmary for Children.

Miss Louisa Palmer, of Wednesbury, has left £100 each to the Wolverhampton General Hospital and the Wednesbury Nursing Institute.

### Passing Events and Topics.

### A Charity Windfall.

Glasgow has found out something original in the way of charity. Two large willow trees in Loch Lomond Park were blown down during a recent gale, and the Corporation has decided to give the trees to the Princess Louise Scottish Hospital for Limbless Sailors and Soldiers at Erskine for the purpose of being used in the making of artificial limbs.

### New Bovril Pictures.

Three new engravings have been issued under the Bovril Bonus Scheme. They are "And they kept the Home Fires Burning," by F. O. Salisbury; "The New Shepherd," by A. J. Elsley; and "In the Heart of the Trossachs," by A. de Branski, jun. They are equal to anything that has been issued in this excellent series.

### Tribute to Lady Medical Officer.

The Education Committee of the East Riding County Council has paid a tribute to Dr. Helen Moffat, the assistant school medical officer, who has now completed more than four years' service under the authority. The committee reports that it cannot speak too highly of the good work she has done under most difficult and trying conditions, and proposes that her salary be increased from £300 to £350 per annum forthwith, and that it be further increased to £400 by two annual increments of £25. The committee suggests that Dr. Moffat shall be responsible for the work of the school medical officer until a new county medical officer of health is appointed as successor to Dr. Wilson, who has resigned.

### Matrons' and Sisters' Appointments.

Alltyr-yn Hospital, Newport, Mon.—Miss Amy Burras has been appointed sister. She was trained at the Cumberland Infirmary, Carlisle, has been sister and night superintendent at the Manchester Sanatorium, Baguley, Cheshire, and sister at the Royal Victoria Infirmary, Newcastle-on-Tyne. She has also done private nursing.

Children's Hospital, Sheffield.—Miss E. Swinden has been appointed sister. She was trained at the Royal Infirmary, Hull, and has been doing private nursing.

Cottage Hospital, Chalfont St. Peter.—Miss C. S. Marshall has been appointed matron. She was trained at Oldham Royal Infirmary, and has held appointments at Bridport Cottage Hospital and Kettering and District General Hospital. She has also been nursing in France.

Cottage Hospital, Colwyn Bay.—Miss L. Howell has been appointed nurse-matron. She was trained at the Royal Gwent Hospital, Newport, and has been sister of the male surgical wards at the Kent and Canterbury Hospital, Canterbury.

District Hospital, Watford, Herts.—Miss A. G. Hughes has been appointed sister. She was trained at the Royal Infirmary, Oldham, and has been sister at the Royal Eye Hospital, Manchester, and at the Infirmary and Dispensary, Rochdale.

General Hospital, Newark-on-Trent.—Miss R. Cudworth has been appointed sister. She was trained at the Royal Infirmary, Oldham, and has been staff nurse at Devonshire Hospital, Buxton. She holds the C.M.B. and I.S.T.M. certificates.

London Homœopathic Hospital, Bloomsbury, W.C.—Miss Edith Annie Eddison, R.R.C., has been appointed matron. She was trained at St. Thomas's Hospital, afterwards being sister-in-charge of a women's surgical ward there. Since 1910 she has been matron of the Royal City of Dublin Hospital. Miss Eddison is a member of the Council of the College of Nursing.

Queen Alexandra's Military Nursing Service for India.—Miss Norah F. M. O'Connor, Miss Constance I. Webb, Miss Elizabeth M. Cameron, and Miss Pauline M. C. Bosanquet have been appointed nursing sisters.

Royal British Orphan Schools, Slough.—Miss A. E. Chevallier has been appointed sister. She was trained at Crumpsall Infirmary, Manchester, and has been superintendent-nurse at Atcham Union Infirmary and at Watford Union Infirmary.

Royal Edinburgh Hospital for Incurables.—Miss Marion W. Smith has been appointed sister. She was trained at Beckett Hospital, Barnsley, has been staff nurse at the Cottage Hospital, Hawick, N.B., and at the Carshalton and District Hospital, and sister of wards and theatre at the Children's Hospital, Sheffield.

**NOTE.—Particulars of Appointments for insertion in this column will be welcomed.**

### Personalia.

Miss Kathleen King (member of the Pharmaceutical Society) has been appointed pharmacist to the Leicester Royal Infirmary to succeed the late Mr. J. W. Cross. Miss King was formerly pharmacist to the Stanley Hospital, Bootle, Liverpool.

Miss Minnie Broad (member of the Pharmaceutical Society), who has been for some years dispenser at the "Eastern" Hospital of the Metropolitan Asylums Board, has been appointed dispenser at the South-Western Hospital under the same Board.

### The Book Market.

LIST OF NEW BOOKS RECEIVED FROM THE FOLLOWING PUBLISHERS :—

H. K. Lewis and Co., Ltd. : "Glaucoma : a Text-book for the Student of Ophthalmology." By Lieutenant-Colonel R. H. Elliott, C.B. 21s. net.

### Papers, Pamphlets, etc.

Report on the Health of Portsmouth for the Year 1916. By A. Mearns Fraser, M.D., Medical Officer of Health.

Third Annual Report of the Lanark District Board of Control.

Messrs. J. and A. Churchill announce the publication of the new (eleventh) edition of "The Practice of Medicine," by Sir Frederick Taylor, Bart., President of the Royal College of Physicians. The work has been subjected to thorough revision, and includes new articles on trench fever, progressive lenticular degeneration, pulmonary embolism, diaphragmatic hernia, so-called soldiers' heart, poisoning by trinitrotoluene, infantilism, renal hæmorrhage, osteogenesis imperfecta, and trench frost-bite. The sections on the ductless or endocrine glands, dysentery, paratyphoid fevers, poliomyelitis, tetanus, hysteria, diseases of muscles, pleurisy, arterial tension, examination of the heart, diseases of the tonsils, diabetes, and beri-beri have been brought up to date, and the number of illustrations in the text has been increased from seventy-one to eighty-five.

A booklet, entitled "Pharmacy and the Public Services," published by the Progressive Pharmacy Club, of which Mr. Herbert Skinner (Great Northern Hospital) is president, very ably deals with the above subject, and should interest all institutional pharmacists. Attention is first concentrated on the Army Pharmaceutical Service and the use that has been made of pharmacists in relief of the medical man, which is claimed to have been less advantageous than it should have been. A skeleton Pharmaceutical Army-Service scheme is very ably shown by diagram, with the head of this proposed Service a Director Pharmaceutical Services, and various grades down to Q.M.S. The duties of a pharmacist at voluntary hospitals and at the large asylums and prisons are summarised.

St. Thomas's Hospital House Appointments.—The following gentlemen have been selected as house officers from Tuesday, January 29, 1918 (subject to the approval of the treasurer).—*Casualty Officers and Resident Anæsthetists* : F. N. V. Dyer, B.A. Cantab., M.R.C.S., L.R.C.P.; J. G. Lawn, B.A. Cantab., M.R.C.S., L.R.C.P.; E. J. Papenfus, M.R.C.S., L.R.C.P.; H. I. Marriner. *Casualty Assistant.*—F. R. G. Hief, B.A. Cantab. *Resident House Physicians.*—F. G. Wood, B.A. Cantab., M.R.C.S., L.R.C.P.; G. H. Sims, M.R.C.S., L.R.C.P.; F. B. Dutton, M.A., M.B., B.Ch. Oxon, M.R.C.S., L.R.C.P.; H. B. Russell, M.R.C.S., L.R.C.P. *Resident House Surgeons*—P. F. Bishop, B.A. Cantab., M.R.C.S., L.R.C.P.; R. M. Humphreys, B.A., M.B., B.Ch. Oxon; C. B. Cohen, B.A. Cantab., M.R.C.S., L.R.C.P.; M. E. Jones, M.R.C.S., L.R.C.P. *House Surgeon to Block 8.*—T. F. M. Dilworth, M.B., B.Ch., B.A.O., N.U.I. *Obstetric House Physicians.*—H. T. Cubbon, B.A. Cantab., M.R.C.S., L.R.C.P.; J. M. Higginton, B.A. Cantab., M.R.C.S., L.R.C.P. *Ophthalmic House Surgeon.*—D. McM. Dickson (M.C.), M.R.C.S., L.R.C.P. *Clinical Assistant (Throat.).*—R. A. Walker.

